# Home Versus Clinic Blood Pressure Monitoring in Women with Hypertensive Disorders of Pregnancy: A Systematic Review and Meta-Analysis

**DOI:** 10.3390/jcm15166195

**Published:** 2026-08-10

**Authors:** Jolene Zhuo Lin Ng, Angela Makris, Renuka Shanmugalingam

**Affiliations:** 1 Department of Renal Medicine, Liverpool Hospital, Sydney, NSW 2170, Australia; angela.makris@health.nsw.gov.au (A.M.); renuka.shanmugalingam@health.nsw.gov.au (R.S.); 2 School of Medicine, Western Sydney University, Campbelltown, NSW 2560, Australia; 3 SWS Clinical School, University of New South Wales, Sydney, NSW 2052, Australia

**Keywords:** home blood pressure monitoring, hypertensive disorders of pregnancy, preeclampsia

## Abstract

**Background/Objectives**: Home blood pressure monitoring (HBPM) is increasingly used in pregnancy, but evidence regarding its safety and clinical utility in women with hypertensive disorders of pregnancy (HDPs) remains limited. We aimed to compare maternal and fetal outcomes and healthcare use with HBPM versus clinic-based blood pressure monitoring. **Methods**: Cochrane, MEDLINE, Embase and PubMed were searched for randomized controlled trials published from January 1970 to December 2022; this review was undertaken to inform guideline development of hypertension in pregnancy in Australia and New Zealand. Two reviewers independently screened studies, extracted data and assessed risk of bias using RoB. Outcomes reported by at least two clinically comparable trials were pooled and heterogeneity was summarized using I^2^. Outcomes reported by a single trial are presented as individual study estimates and were not meta-analyzed. Certainty of evidence was assessed using GRADE. **Results**: Four randomized trials involving 3533 participants were included. HBPM was not associated with a statistically significant difference in preeclampsia, adverse maternal composite outcomes, severe hypertension (≥160/110 mmHg), emergency delivery for hypertension, stillbirth, small-for-gestational-age birth or neonatal mortality. A single trial also found no statistically significant difference in preterm birth before 34 weeks. Serious fetal outcomes were uncommon and confidence intervals were wide. In one pilot trial, HBPM was associated with fewer antenatal visits and a longer duration of blood pressure monitoring. **Conclusions**: In structured care pathways with clinician oversight and predefined escalation protocols, HBPM was not associated with evidence of increased maternal or fetal risk and may be a useful adjunct to standard antenatal care. The small number of United Kingdom trials, sparse serious events and limited evidence in established or severe preeclampsia preclude conclusions of equivalence or definitive safety.

## 1. Introduction

Hypertension affects up to 10% of all pregnancies worldwide with an estimated incidence of 18.1 million cases annually [1,2]. It is a major contributor to outpatient clinic visits, hospitalisations, perinatal morbidity, and mortality [3]. Hypertensive disorders of pregnancy (HDPs) refer to gestational hypertension, superimposed preeclampsia, preeclampsia and chronic hypertension [4]. The introduction of antihypertensives since the 1960s has significantly reduced perinatal morbidity and mortality with the optimal target blood pressure threshold being revised and redefined [5].

Accurate diagnosis and management of HDPs depend on reliable blood pressure measurement. HBPM is the self-measurement of blood pressure outside the clinic using an automated upper-arm device, undertaken within a care pathway that specifies measurement technique, review of results and escalation thresholds. Potential advantages include more frequent measurements, identification of white-coat or masked hypertension and fewer clinic attendances; however, these benefits depend on device validation, patient training and timely clinical review [6].

Despite increasing use, evidence-based recommendations for HBPM in pregnancy remain limited. Important uncertainties include the validity of devices in pregnancy, measurement technique, adherence, escalation pathways and the populations in whom HBPM can be used safely. The International Society for the Study of Hypertension in Pregnancy supports out-of-office monitoring as an adjunct in selected women, but evidence for maternal and perinatal outcomes remains sparse [7].

## 2. Aims

This review synthesized the evidence available to the development group for the Society of Obstetric Medicine of Australia and New Zealand (SOMANZ) Hypertension in Pregnancy Guideline 2023. The original search therefore included evidence published to December 2022.

The primary objective was to compare adverse maternal and fetal outcomes in pregnant women managed using HBPM within a structured clinical pathway versus standard clinic-based blood pressure monitoring.

The main outcomes were preeclampsia, adverse maternal composite outcomes, severe hypertension (≥160/110 mmHg), emergency delivery for hypertension, stillbirth, preterm delivery before 34 weeks, small-for-gestational-age birth (<10th centile) and neonatal mortality. Secondary outcomes were the number of antenatal visits, the number of weeks during which blood pressure monitoring was performed and time to clinically confirmed hypertension.

## 3. Methodology

A systematic review and meta-analysis of randomized controlled trials (RCTs) addressed the following prespecified PICO question: in pregnant women at risk of or with HDPs (population), does HBPM (intervention), compared with clinic-based blood pressure monitoring (comparison), affect maternal and fetal clinical outcomes and healthcare use (outcomes)?

The review is reported in accordance with the PRISMA 2020 statement and PRISMA guidance for reporting literature searches [8,9]. The review protocol was not prospectively registered; the review was conducted as part of a broader guideline-development process. A completed PRISMA 2020 checklist is provided in Supplementary File S2.

### 3.1. Literature Search and Selection

Cochrane CENTRAL, MEDLINE, Embase and PubMed were searched for records published from January 1970 to December 2022. Search concepts covered HDPs, HBPM and prespecified maternal and fetal outcomes. Subject headings and free-text synonyms were combined using Boolean operators. The complete database-specific strategies, limits and search dates are provided in Supplementary File S1.

Two reviewers independently screened titles and abstracts and then assessed potentially eligible full texts using predetermined population, intervention, comparator and outcome criteria. RCTs comparing structured HBPM with clinic-based monitoring were eligible for quantitative synthesis. Observational studies and other study designs were identified by the search but were excluded during full-text selection under the study-design criterion. Disagreements were resolved by discussion or a third reviewer. Studies involving non-pregnant or non-human subjects, non-English publications, unpublished data, case reports and case series were excluded.

### 3.2. Risk of Bias Assessment

Risk of bias assessment for the selected randomized controlled studies were performed using the Cochrane risk of bias tool for randomized trials (RoB) [10]. Multiple signalling questions within each of the five independent domains were answered by two independent reviewers, culminating as an overall risk of bias judgement rating allocated for each domain. Risk of bias ratings were discussed among the two authors and when disagreements arose, a review from a third author was sought to achieve consensus.

### 3.3. Data Collection and Analysis

Definitions of maternal and fetal outcomes are detailed in Table 1. Secondary outcomes assessed include number of antenatal clinic visits, frequency of blood pressure measurements (in weeks) and time to diagnosis of clinically confirmed hypertension.

Data were extracted independently by two authors and checked for consistency. Where there were disagreements, a review by a third author was sought. Data extracted was entered into Review Manager 5.4.1 (Cochrane Collaboration, Oxford, UK) to facilitate meta-analysis and forest plots.

Dichotomous outcomes were expressed as risk ratios (RRs) with 95% confidence intervals (CIs), and continuous outcomes as mean differences (MDs) with 95% CIs. Where at least two clinically comparable trials reported an outcome, estimates were pooled using Mantel–Haenszel random-effects models for dichotomous outcomes or inverse-variance random-effects models for continuous outcomes. Statistical heterogeneity was summarized using the χ^2^ test and I^2^ statistic. Outcomes reported by only one trial were not meta-analyzed; the individual trial estimate and 95% CI were reported descriptively, and I^2^ was not applicable.

Certainty of evidence for each outcome was assessed using GRADEpro and the GRADE domains of risk of bias, inconsistency, indirectness, imprecision and publication bias, resulting in a rating of high, moderate, low or very low certainty.

## 4. Results

**Figure 1:**
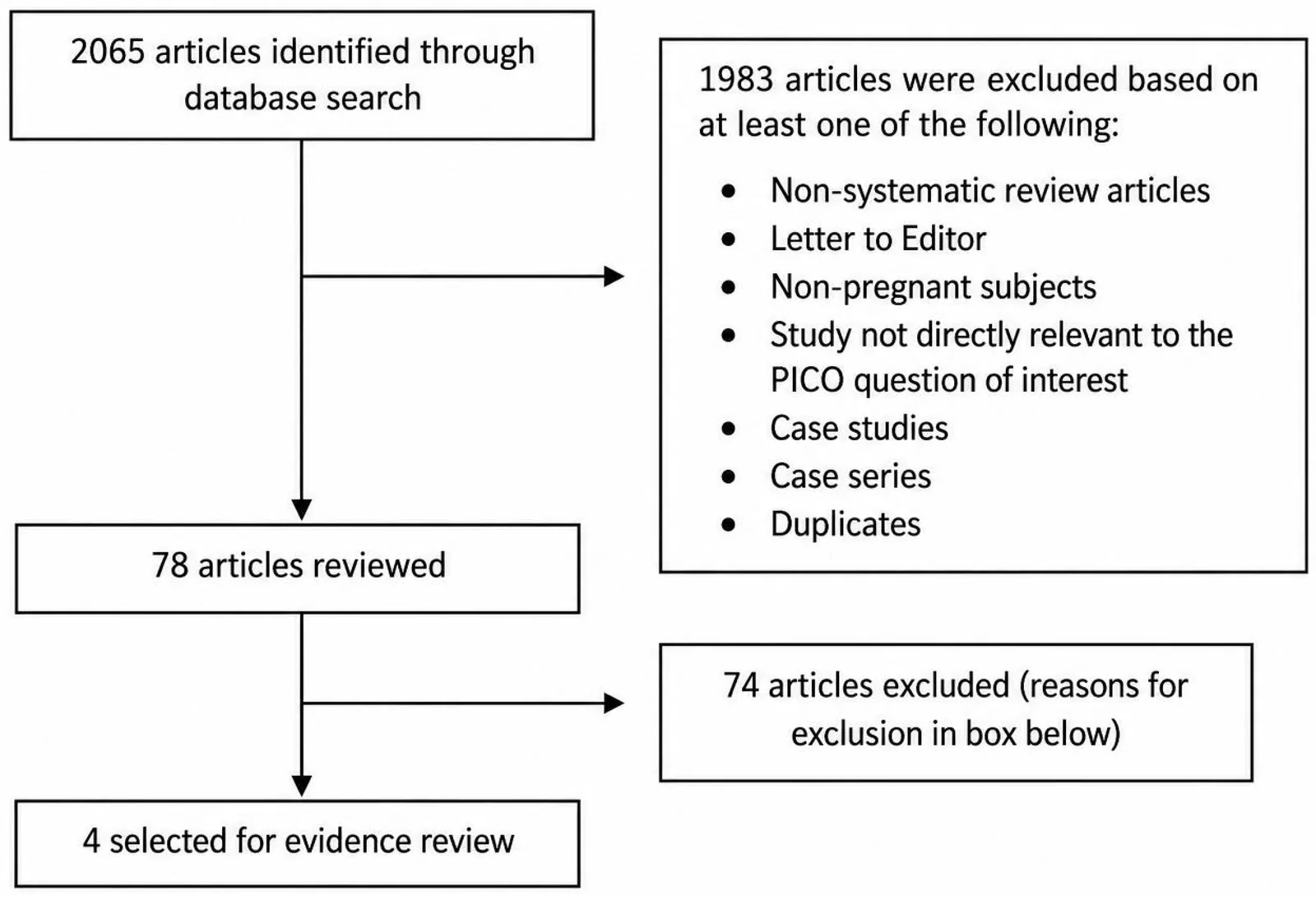
Study flow diagram—selection process for included studies.

The database search identified 2065 records. The selection process is summarized in Figure 1 and the full-text exclusion list is provided in Supplementary File S1.

After duplicate removal and title/abstract screening, potentially eligible reports were assessed in full text by two independent reviewers. Disagreements were resolved by discussion or a third reviewer.

Full-text reports were excluded principally because they assessed device accuracy rather than clinical outcomes, used an ineligible study design, or did not address the prespecified PICO question.

Four RCTs involving 3533 participants were included in the review and quantitative or structured synthesis (Figure 1). Study characteristics are summarized in Table 2.

Risk of bias was judged as low for the randomization, allocation concealment, missing outcome data and selective reporting across the four trials. Because participants and clinical teams could not be blinded to HBPM, the domain relating to deviations from intended interventions was judged as high-risk or as having some concerns, as appropriate.

**Figure 2:**
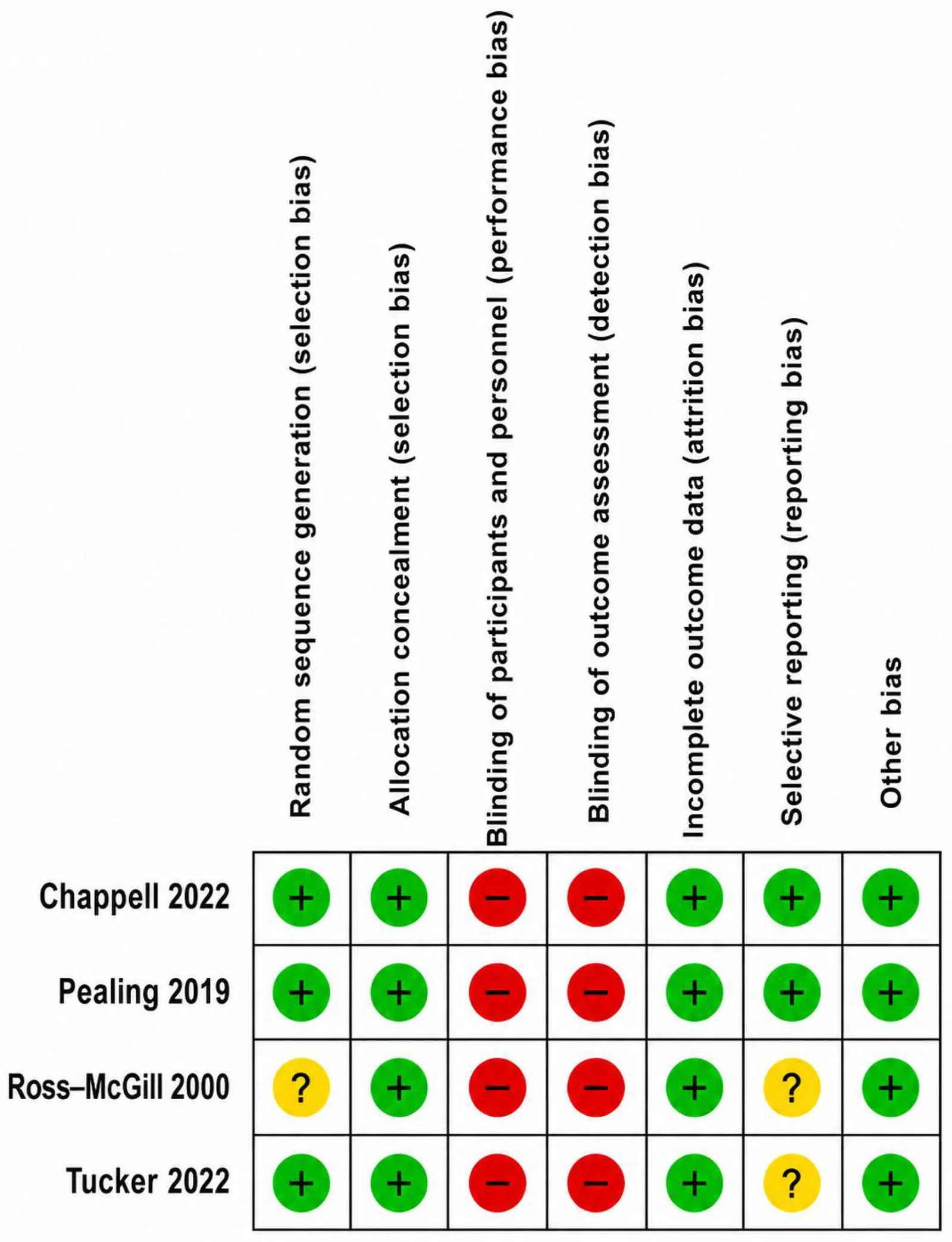
Risk of bias summary. Included randomized controlled trials: Chappell et al. (2022) [12], Pealing et al. (2019) [13], Ross-McGill et al. (2000) [14], Tucker et al. (2022) [11]. Green (+), low risk of bias; red (−), high risk of bias; yellow (?), unclear risk of bias.

Figure 2 summarizes domain-level judgements as low-risk (green), some concerns (yellow) or high-risk (red).

Across the seven outcomes pooled from at least two trials, no statistically significant between-group differences were observed. One additional prespecified outcome, preterm delivery before 34 weeks, was reported by a single trial and was therefore not meta-analyzed. Heterogeneity was low for the pooled outcomes (I^2^ 0–21%).

### 4.1. Main Outcomes

#### 4.1.1. Maternal Outcomes

**Preeclampsia:**

**Figure 3:**
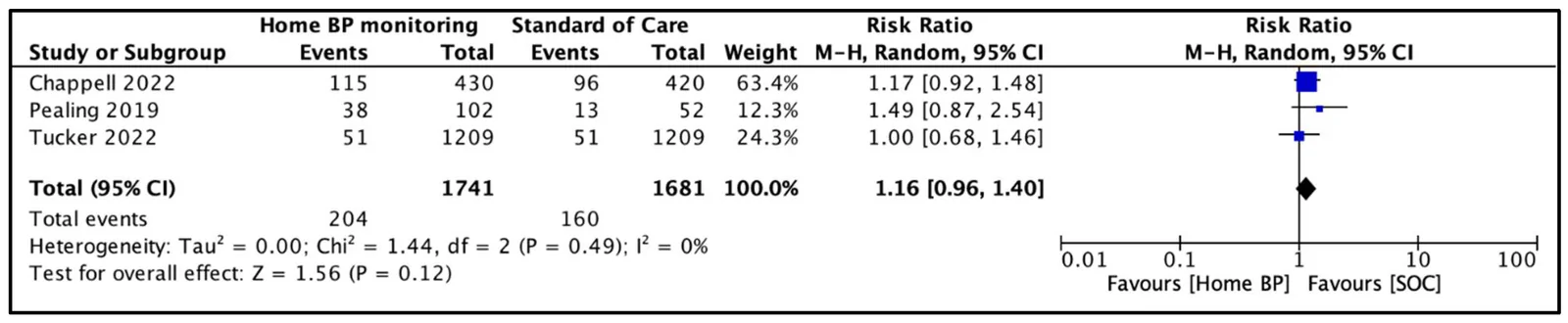
Rates of preeclampsia. Forest plot comparing home blood pressure monitoring (HBPM) with standard of care (SOC). Blue squares represent individual study risk ratios, with square size proportional to study weight; horizontal lines represent 95% confidence intervals (CIs). The black diamond represents the pooled risk ratio and 95% CI. The vertical line indicates the line of no effect (RR = 1). Included randomized controlled trials: Chappell et al. (2022) [12], Pealing et al. (2019) [13], Tucker et al. (2022) [11].

Preeclampsia occurred in 204 of 1741 participants (11.7%) in the HBPM group and 160 of 1681 (9.5%) in the clinic-monitoring group. The pooled estimate did not show a statistically significant difference (RR 1.16, 95% CI 0.96–1.40; *p* = 0.12; I^2^ = 0%; Figure 3).

**Adverse maternal composite outcomes:**

**Figure 4:**
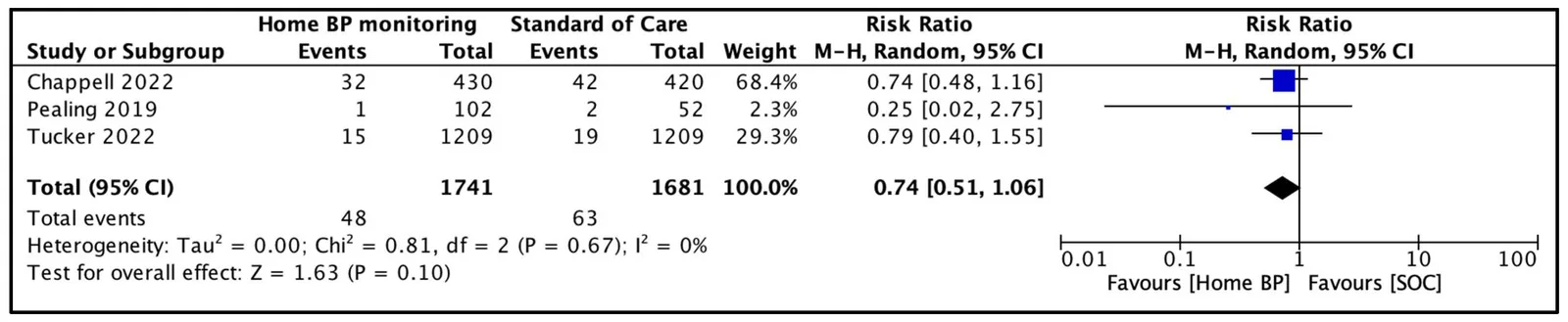
Combined adverse maternal composite outcomes. Forest plot comparing HBPM with SOC. Blue squares represent individual study risk ratios, with square size proportional to study weight; horizontal lines represent 95% CIs. The black diamond represents the pooled risk ratio and 95% CI. The vertical line indicates the line of no effect (RR = 1). Included randomized controlled trials: Chappell et al. (2022) [12], Pealing et al. (2019) [13], Tucker et al. (2022) [11].

Adverse maternal composite outcomes occurred in 48 of 1741 participants (2.8%) in the HBPM group and 63 of 1681 (3.7%) in the clinic-monitoring group, with no statistically significant difference (RR 0.74, 95% CI 0.51–1.06; *p* = 0.10; I^2^ = 0%; Figure 4).

**Severe hypertension (BP ≥ 160/110 mmHg):**

**Figure 5:**
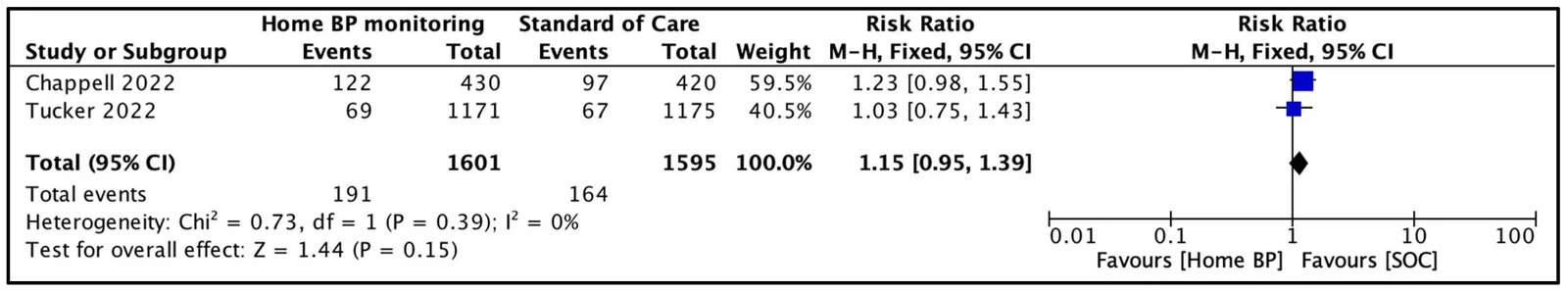
Rates of severe hypertension. Forest plot comparing HBPM with SOC. Blue squares represent individual study risk ratios, with square size proportional to study weight; horizontal lines represent 95% CIs. The black diamond represents the pooled risk ratio and 95% CI. The vertical line indicates the line of no effect (RR = 1). Included randomized controlled trials: Chappell et al. (2022) [12], Tucker et al. (2022) [11].

Severe hypertension occurred in 191 of 1601 participants (11.9%) in the HBPM group and 164 of 1595 (10.3%) in the clinic-monitoring group. The pooled estimate was not statistically significant (RR 1.15, 95% CI 0.95–1.39; *p* = 0.15; I^2^ = 0%; Figure 5).

**Emergency delivery for hypertension:**

**Figure 6:**
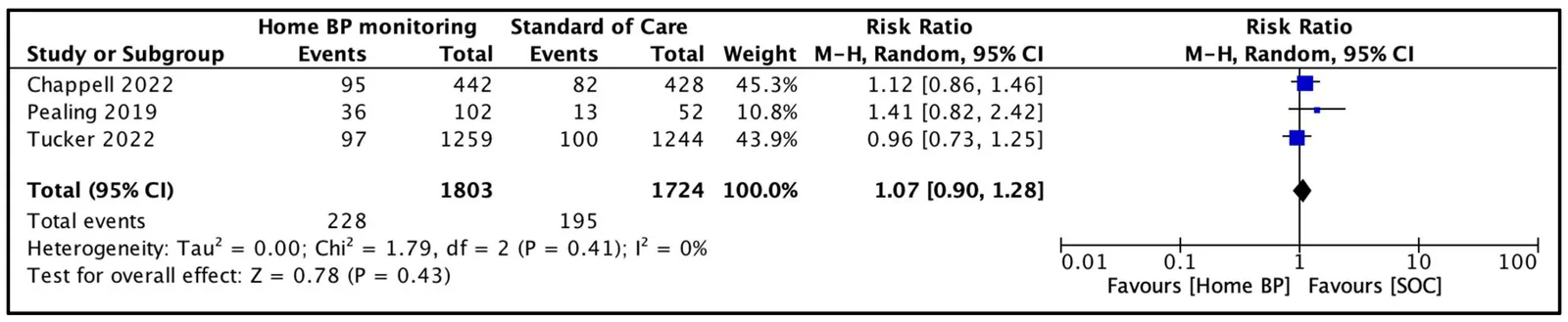
Rates of emergency delivery for hypertension. Forest plot comparing HBPM with SOC. Blue squares represent individual study risk ratios, with square size proportional to study weight; horizontal lines represent 95% CIs. The black diamond represents the pooled risk ratio and 95% CI. The vertical line indicates the line of no effect (RR = 1). Included randomized controlled trials: Chappell et al. (2022) [12], Pealing et al. (2019) [13], Tucker et al. (2022) [11].

Emergency delivery for hypertension occurred in 228 of 1803 participants (12.6%) in the HBPM group and 195 of 1724 (11.3%) in the clinic-monitoring group, with no statistically significant difference (RR 1.07, 95% CI 0.90–1.28; *p* = 0.43; I^2^ = 0%; Figure 6).

#### 4.1.2. Fetal Outcomes

**Stillbirth:**

**Figure 7:**
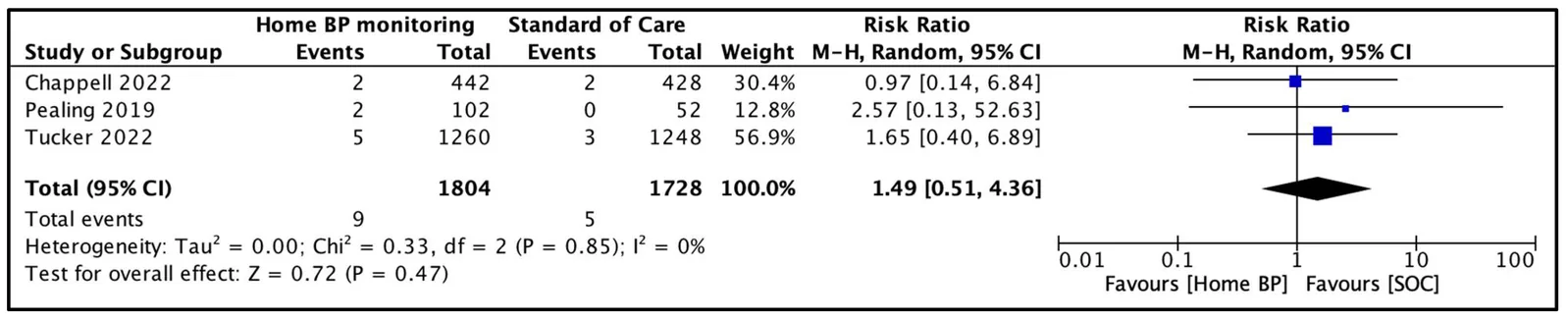
Rates of stillbirth. Included randomized controlled trials: Chappell et al. (2022) [12], Pealing et al. (2019) [13], Tucker et al. (2022) [11].

Stillbirth occurred in 9 of 1804 pregnancies (0.50%) in the HBPM group and 5 of 1728 (0.29%) in the clinic-monitoring group. The estimate was imprecise and did not show a statistically significant difference (RR 1.49, 95% CI 0.51–4.36; *p* = 0.47; I^2^ = 0%; Figure 7).

**Preterm delivery (<34 weeks):**

One trial reported preterm delivery before 34 weeks in 9 of 102 participants (8.8%) in the HBPM group and 4 of 52 (7.7%) in the clinic-monitoring group (RR 1.15, 95% CI 0.37–3.55; *p* = 0.81). Because only one trial contributed data, no pooled meta-analysis or heterogeneity estimate was calculated.

**Small for gestational age (<10th centile):**

**Figure 8:**
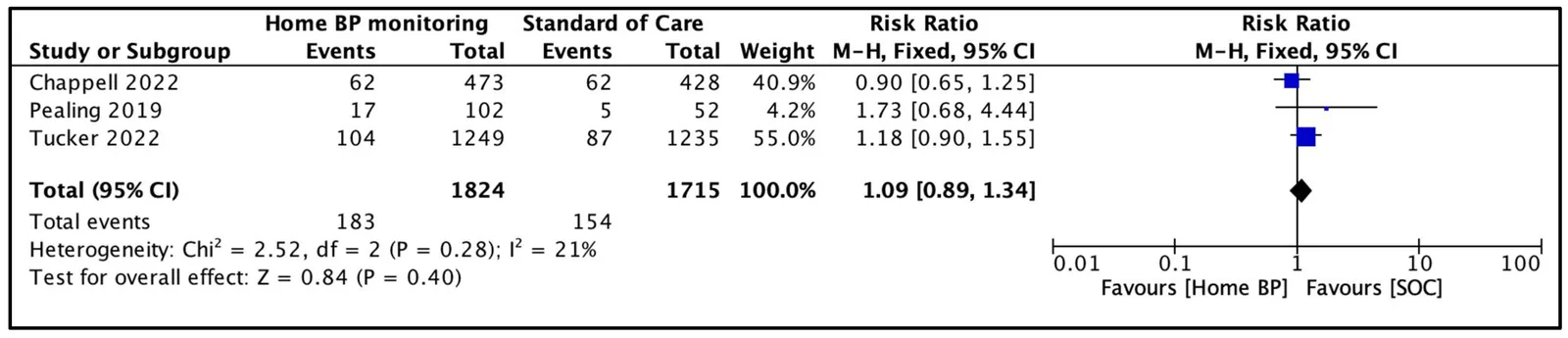
Rates of small-for-gestational-age babies. Included randomized controlled trials: Chappell et al. (2022) [12], Pealing et al. (2019) [13], Tucker et al. (2022) [11].

Small-for-gestational-age birth occurred in 183 of 1824 infants (10.0%) in the HBPM group and 154 of 1715 (9.0%) in the clinic-monitoring group. The pooled estimate was not statistically significant (RR 1.09, 95% CI 0.89–1.34; *p* = 0.40; I^2^ = 21%; Figure 8).

**Neonatal mortality:**

**Figure 9:**
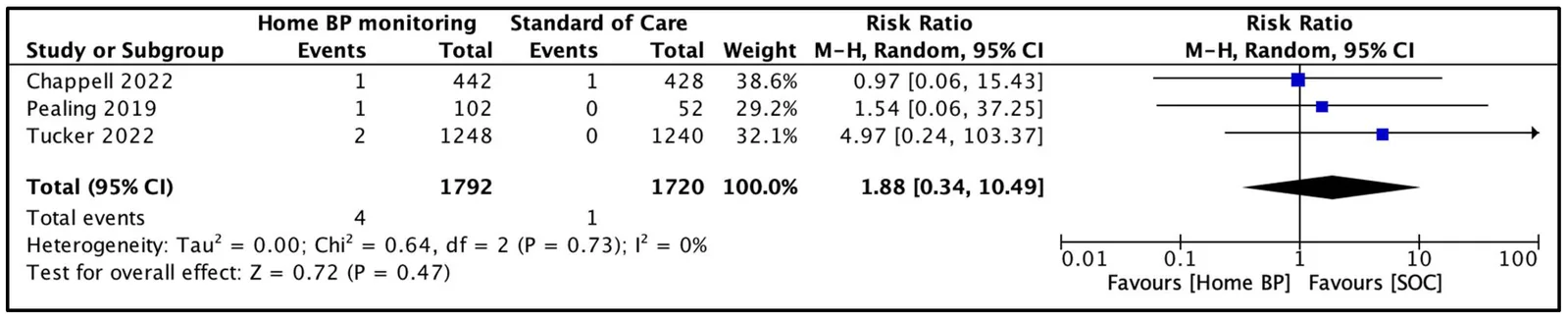
Rates of neonatal mortality. Included randomized controlled trials: Chappell et al. (2022) [12], Pealing et al. (2019) [13], Tucker et al. (2022) [11].

Neonatal death occurred in 4 of 1792 liveborn infants (0.22%) in the HBPM group and 1 of 1720 (0.06%) in the clinic-monitoring group. The estimate was highly imprecise and did not show a statistically significant difference (RR 1.88, 95% CI 0.34–10.49; *p* = 0.47; I^2^ = 0%; Figure 9).

### 4.2. Secondary Outcomes

#### 4.2.1. Number of Antenatal Visits

One trial reported fewer antenatal visits with HBPM than with clinic monitoring (mean 4.5 versus 7.4 visits; MD −2.90 visits, 95% CI −3.86 to −1.94; *p* < 0.00001). This was a single-study estimate and was not meta-analyzed.

#### 4.2.2. Number of Weeks During Which Blood Pressure Monitoring Was Performed

The same trial reported that blood pressure monitoring was undertaken over a longer period after recruitment with HBPM than with clinic monitoring (mean 10.0 versus 6.9 weeks; MD 3.10 weeks, 95% CI 2.09–4.11; *p* < 0.00001). This outcome represents the duration of monitoring rather than the number of individual readings and was not meta-analyzed.

#### 4.2.3. Time to Diagnosis of Clinically Confirmed Hypertension (Days)

One trial found no statistically significant difference in time to clinically confirmed hypertension between HBPM and clinic-based monitoring (MD −1.90 days, 95% CI −4.51 to 0.71; *p* = 0.15). This was a single-study estimate and was not meta-analyzed.

## 5. Discussion

In this review, HBPM delivered within structured care pathways was not associated with a statistically significant increase in the maternal or fetal outcomes examined. This finding should not be interpreted as proof of equivalence or definitive safety: only four RCTs were available, serious outcomes were uncommon and confidence intervals for stillbirth and neonatal mortality were wide. The evidence therefore supports HBPM as a potential adjunct to, rather than a replacement for, standard antenatal assessment in carefully selected women.

The findings are broadly consistent with previous reviews. Albadrani et al. (2023) reported no increase in maternal, fetal or neonatal adverse outcomes with HBPM, although that review also included non-randomized designs [15]. Yeh et al. (2022) similarly found no clear adverse association across one RCT and two observational studies and reported generally high patient satisfaction [16]. The narrower inclusion of RCTs in the present review reduces confounding but also limits the amount and precision of available evidence.

International and local guidelines increasingly recognize HBPM as an adjunct to care [4,7]. The ISSHP recommendations support out-of-office monitoring after an elevated clinic blood pressure when preeclampsia has not been established, while the SOMANZ guideline supports HBPM in chronic or gestational hypertension without replacing the minimum recommended frequency of antenatal review. These recommendations align with the context of the included trials, all of which incorporated clinician oversight, patient instructions and predefined thresholds for escalation. The findings should not be extrapolated to completely unsupervised monitoring or to women with established severe preeclampsia, for whom evidence remains limited.

Real-world implementation, however, depends on more than provision of a monitor. Participants must be able to use a device validated for pregnancy, select the correct cuff size, follow a standardized measurement technique, record or transmit readings reliably and act on abnormal values. Adherence may decline over time, and inaccurate technique or delayed escalation could create false reassurance. Digital health literacy, language, connectivity, smartphone access, affordability and the capacity of clinical teams to review readings may influence both effectiveness and equity. Services should therefore include device validation, practical training, written escalation instructions, technical support and an alternative pathway for women unable to engage safely with digital monitoring.

All included trials were conducted in the United Kingdom, within a health system providing structured antenatal care and, in the larger trials, digital telemonitoring support. Generalizability may be limited in settings with different thresholds for assessment or admission, less frequent clinical contact, different population characteristics, limited access to validated devices or reliable connectivity, and fewer resources for timely review of transmitted readings. Conversely, HBPM may offer particular value in rural or geographically dispersed populations if the clinical infrastructure required for supervision and escalation is available.

Strengths of this review include independent screening and extraction, inclusion of randomized trials, RoB assessment and GRADE evaluation. Important limitations are the small number of trials, sparse serious outcomes, wide confidence intervals, geographic concentration in the United Kingdom and limited evidence in established preeclampsia. Formal assessment of publication bias was not informative with only four included trials and was therefore not performed. The search was originally completed for a 2023 guideline process and ended in December 2022; this limits the currency of the journal review unless the search is updated. Further studies should evaluate diverse populations, patient-reported outcomes, adherence, health-system costs and standardized training and escalation pathways.

## 6. Conclusions

Home blood pressure monitoring was not associated with evidence of increased maternal or fetal risk in the available RCTs, but the evidence is insufficient to establish equivalence or definitive safety. HBPM may be a useful adjunct to standard antenatal care for carefully selected women when a pregnancy-validated device, patient education, clinician oversight and clear escalation protocols are provided. It should not replace recommended antenatal review, and further evidence is required in severe preeclampsia and in healthcare settings outside the United Kingdom.

## Figures and Tables

**Table 1:** Definition of maternal and fetal outcomes.

Definition of Maternal Outcomes	
Preeclampsia	New onset of hypertension (systolic blood pressure ≥140 mmHg and/or diastolic blood pressure ≥90 mmHg) after 20 weeks gestation accompanied by ≥1 new onset organ involvement (renal, liver, hematological, neurological, pulmonary or uteroplacental dysfunction)
Adverse maternal composite outcomes	One or more of the following: eclampsia, intracranial hemorrhage, transient ischaemic attack or infarct, HELLP (haemolysis, elevated liver enzymes, low platelets) syndrome, liver involvement (ALT or AST > 70 U/L), pulmonary edema, kidney involvement (serum creatinine >90 μmol/L), and hematological involvement (platelets <100 × 10^9^ /L) or maternal death
Severe hypertension (≥160/110 mmHg)	Systolic blood pressure ≥160 mmHg and/or diastolic blood pressure ≥110 mmHg, confirmed on repeat measurement
Emergency delivery for hypertension	Emergency delivery performed within 48 h (with primary indication for delivery to be severe hypertension)
Admission into ICU	Any maternal admission to intensive care unit during antenatal or postpartum period
Need for IV antihypertensive therapy week before delivery	Administration of IV antihypertensive medications during week prior to delivery
Definition of Fetal Outcomes	
Stillbirth	Fetal death prior to birth of a baby of ≥20 completed weeks of gestation or of ≥400 g birthweight
Preterm delivery (<34 weeks)	Any birth before 34 completed weeks of pregnancy
Small for gestational age (<10th centile)	Infants with birthweight below the 10th percentile for their gestational age and sex
Small for gestational age (<3rd centile)	Infants with birthweight below the 3rd percentile for their gestational age and sex
Neonatal mortality	Death of liveborn baby of ≥20 completed weeks of gestation or of ≥400 g birthweight within 28 days of birth
NICU admission	Any neonatal intensive care unit admission required

**Table 2:** Characteristics of included studies.

Study & Type	Country	Total Patients	Definition of HBPM	Gestation	Duration of Study
Tucker et al. (RCT) 2022 [11]	United Kingdom	2441	HBPM with readings transmitted through a mobile-phone platform, with an optional paper diary (Microlife WatchBP Home).	Mean gestation of 20 weeks	November 2018 to October 2019
Chappell et al. (RCT) 2022 [12]	United Kingdom	850	HBPM with readings transmitted through a mobile-phone platform, with an optional paper diary (Microlife WatchBP Home).	Mean gestation of 20 weeks	November 2018 to September 2019
Pealing et al. (RCT) 2019 [13]	United Kingdom	162	HBPM with readings recorded using a paper diary, SMS platform or mobile application (Microlife WatchBP Home).	Mean gestation between 14.9 and 16.6 weeks	December 2015 to December 2017
Ross-McGill et al. (RCT) 2000 [14]	United Kingdom	80	A portable blood pressure machine with results recorded in a paper diary (Omron).	Mean gestation 24–28 weeks	Study duration unclear

## Data Availability

The data supporting the findings of this systematic review were derived from the published studies cited in the article. The extracted data used for the analyses are available from the corresponding author upon reasonable request. The PICO framework, search strategies and study-selection materials are provided in Supplementary File S1.

## Supplementary Materials

The following supporting information can be downloaded at https://www.mdpi.com/article/10.3390/jcm15166195/s1, Supplementary File S1: PICO Framework, Search Strategies and Study Selection; Supplementary File S2: PRISMA 2020 checklist.
