# Supplementary material for: Home Versus Clinic Blood Pressure Monitoring in Women with Hypertensive Disorders of Pregnancy: A Systematic Review and Meta-Analysis
Source: J Clin Med. 2026 Aug 10;15(16):6195. doi: 10.3390/jcm15166195 (PMC13513794; doi:10.3390/jcm15166195)
Supplement: Supplementary file 1 [file jcm-15-06195-s001.zip › Supplementary File S2.pdf]

PRISMA 2020 Checklist

Home Versus Clinic Blood Pressure Monitoring in Women with Hypertensive Disorders of Pregnancy: A Systematic Review and Meta-Analysis

Topic	Item	PRISMA 2020 checklist item	Location where item is reported / author note
Title	1	Identify the report as a systematic review.	Title: "Home Versus Clinic Blood Pressure Monitoring in Women with Hypertensive Disorders of Pregnancy: A Systematic Review and Meta-Analysis."
Abstract	2	See the PRISMA 2020 for Abstracts checklist.	Abstract (structured: Background, Aims, Methods, Results and Conclusions).
Rationale	3	Describe the rationale for the review in the context of existing knowledge.	Introduction, paragraphs 1–3.
Objectives	4	Provide an explicit statement of the objective(s) or question(s) the review addresses.	Section 2, Aims; primary objective and prespecified primary/secondary outcomes.
Eligibility criteria	5	Specify the inclusion and exclusion criteria for the review and how studies were grouped for the syntheses.	Section 3.1, Literature Search and Selection; Supplementary Appendix S1, S1 PICO Framework. Grouping for synthesis described in Section 3.3.
Information sources	6	Specify all databases, registers, websites, organisations, reference lists and other sources searched or consulted to identify studies. Specify the date when each source was last searched or consulted.	Section 3.1 names Cochrane CENTRAL, MEDLINE, Embase and PubMed; Supplementary Appendix S1, S2–S4.
Search strategy	7	Present the full search strategies for all databases, registers and websites, including any filters and limits used.	Supplementary Appendix S1, S3–S4.
Selection process	8	Specify the methods used to decide whether a study met the inclusion criteria, including how many reviewers screened each record and report retrieved, whether they worked independently, and, if applicable, details of automation tools used.	Section 3.1: two reviewers independently screened titles/abstracts and full texts; disagreements resolved by discussion or a third reviewer. No automation tools reported.
Data collection process	9	Specify the methods used to collect data from reports, including how many reviewers collected data from each report, whether they worked independently, processes for obtaining or confirming data, and, if applicable, details of automation tools used.	Section 3.3: data independently extracted by two authors, checked for consistency, with third-author review for disagreements. Methods for contacting investigators or confirming missing data are not reported as not performed.
Data items	10a	List and define all outcomes for which data were sought. Specify whether all results compatible with each outcome domain in each study were sought and, if not, the methods used to decide which results to collect.	Section 2, Aims; Section 3.3; Table 1 defines maternal and fetal outcomes.
Data items	10b	List and define all other variables for which data were sought. Describe any assumptions made about missing or unclear information.	Table 2 reports country, sample size, HBPM method/device, gestation and study duration. Assumptions regarding missing or unclear information are not explicitly described.
Study risk of bias assessment	11	Specify the methods used to assess risk of bias in the included studies, including details of the tool(s) used, how many reviewers assessed each study and whether they worked independently, and, if applicable, details of automation tools used.	Section 3.2: Cochrane RoB 2; two independent reviewers; disagreements discussed and referred to a third author. No automation tools reported.
Effect measures	12	Specify for each outcome the effect measure(s) used in the synthesis or presentation of results.	Section 3.3: risk ratios with 95% CIs for dichotomous outcomes; mean differences with 95% CIs for continuous outcomes.
Synthesis methods	13a	Describe the processes used to decide which studies were eligible for each synthesis.	Section 3.1 and Section 3.3: eligible RCTs were grouped by clinically comparable outcomes; outcomes reported by at least two comparable trials were pooled, while single-trial outcomes were reported separately.
Synthesis methods	13b	Describe any methods required to prepare the data for presentation or synthesis, such as handling of missing summary statistics or data conversions.	No explicit data-preparation, conversion or imputation methods are described.
Synthesis methods	13c	Describe any methods used to tabulate or visually display results of individual studies and syntheses.	Section 3.3: Review Manager 5.4.1 used for meta-analysis and forest plots; Tables 1–2 and Figures 3–9 present results.
Synthesis methods	13d	Describe any methods used to synthesize results and provide a rationale for the choice(s). If meta-analysis was performed, describe the model(s), method(s) to identify heterogeneity, and software package(s) used.	Section 3.3: Mantel–Haenszel random-effects models for dichotomous outcomes; inverse-variance random-effects models for continuous outcomes; χ^2 and I^2 for heterogeneity; Review Manager 5.4.1.
Synthesis methods	13e	Describe any methods used to explore possible causes of heterogeneity among study results.	No subgroup analysis, meta-regression or other exploration of heterogeneity is reported. Heterogeneity was low and only four trials were included.
Synthesis methods	13f	Describe any sensitivity analyses conducted to assess robustness of the synthesized results.	No sensitivity analyses are reported.
Reporting bias assessment	14	Describe any methods used to assess risk of bias due to missing results in a synthesis.	Discussion states formal publication-bias assessment was not informative with four trials and was not performed; no separate methods statement is provided.
Certainty assessment	15	Describe any methods used to assess certainty (or confidence) in the body of evidence for an outcome.	Section 3.3: GRADEpro and the GRADE domains of risk of bias, inconsistency, indirectness, imprecision and publication bias; certainty rated high, moderate, low or very low.

Topic	Item	PRISMA 2020 checklist item	Location where item is reported / author note
Study selection	16a	Describe the results of the search and selection process, from the number of records identified in the search to the number of studies included in the review, ideally using a flow diagram.	Section 4 and Figure 1; Supplementary Appendix S1, S5.
Study selection	16b	Cite studies that might appear to meet the inclusion criteria, but which were excluded, and explain why they were excluded.	Supplementary Appendix S1, S7 provides a full-text exclusion list and reasons.
Study characteristics	17	Cite each included study and present its characteristics.	Section 4, Table 2; included trials cited as Tucker et al., Chappell et al., Pealing et al. and Ross-McGill et al.
Risk of bias in studies	18	Present assessments of risk of bias for each included study.	Section 4 and Figure 2, Risk of Bias Summary. Domain-level judgements are described; ensure the final figure clearly presents each study's judgement.
Results of individual studies	19	For all outcomes, present for each study: summary statistics for each group and an effect estimate and its precision, ideally using structured tables or plots.	Sections 4.1–4.2 and Figures 3–9. Single-trial outcomes are reported with group data, effect estimates and 95% CIs.
Results of syntheses	20a	For each synthesis, briefly summarise the characteristics and risk of bias among contributing studies.	Section 4, Table 2 and Figure 2 provide characteristics and risk-of-bias information; the narrative synthesis does not repeat these details for every individual outcome.
Results of syntheses	20b	Present results of all statistical syntheses conducted. If meta-analysis was done, present for each the summary estimate and its precision and measures of statistical heterogeneity. If comparing groups, describe the direction of the effect.	Sections 4.1–4.2; Figures 3–9. RRs or MDs, 95% CIs, P values and I^2 are reported; direction is described.
Results of syntheses	20c	Present results of all investigations of possible causes of heterogeneity among study results.	No investigations of heterogeneity were performed.
Results of syntheses	20d	Present results of all sensitivity analyses conducted to assess robustness of the synthesized results.	No sensitivity analyses were performed.
Reporting biases	21	Present assessments of risk of bias due to missing results for each synthesis assessed.	Formal assessment was not performed because only four trials were included; this is acknowledged in the Discussion section.
Certainty of evidence	22	Present assessments of certainty (or confidence) in the body of evidence for each outcome assessed.	GRADE was performed as discussed in Methods section.
Discussion	23a	Provide a general interpretation of the results in the context of other evidence.	Section 5, Discussion, paragraphs 1–3.
Discussion	23b	Discuss any limitations of the evidence included in the review.	Section 5: four RCTs, sparse serious outcomes, wide CIs, UK-only setting and limited evidence in severe preeclampsia.
Discussion	23c	Discuss any limitations of the review processes used.	Section 5 acknowledges the search ended in December 2022 and publication bias could not be formally assessed.
Discussion	23d	Discuss implications of the results for practice, policy and future research.	Section 5 and Section 6: HBPM as an adjunct within structured care; implementation requirements; need for diverse populations, patient-reported outcomes, adherence, costs and standardised pathways.
Registration and protocol	24a	Provide registration information for the review, including register name and registration number, or state that the review was not registered.	Section 3 states that the protocol was not prospectively registered.
Registration and protocol	24b	Indicate where the review protocol can be accessed, or state that a protocol was not prepared.	A protocol was not prepared.
Registration and protocol	24c	Describe and explain any amendments to information provided at registration or in the protocol.	Not applicable.
Support	25	Describe sources of financial or non-financial support for the review, and the role of the funders or sponsors in the review.	No external funding was received – included in Funding section.
Competing interests	26	Declare any competing interests of review authors.	The authors declare no conflict of interest – included in Conflicts of Interest section.
Availability of data, code and other materials	27	Report which of the following are publicly available and where they can be found: template data collection forms; data extracted from included studies; data used for all analyses; analytic code; and any other materials used in the review.	Supplementary Appendix S1 contains PICO/search/study-selection materials.
