# Supplementary material for: Home Versus Clinic Blood Pressure Monitoring in Women with Hypertensive Disorders of Pregnancy: A Systematic Review and Meta-Analysis
Source: J Clin Med. 2026 Aug 10;15(16):6195. doi: 10.3390/jcm15166195 (PMC13513794; doi:10.3390/jcm15166195)
Supplement: Supplementary file 1 [file jcm-15-06195-s001.zip › Supplementary File S1.pdf]

Supplementary File S1

PICO Framework, Search Strategies and Study Selection

Home Versus Clinic Blood Pressure Monitoring in Women with Hypertensive Disorders of Pregnancy

Is home blood pressure monitoring (with an automated home monitoring machine) a safe alternative to clinical blood pressure monitoring for follow up/monitoring of women with hypertension in pregnancy?

Review Question and PICO Framework

**Patient**	Pregnant women with hypertension
**Intervention**	Home blood pressure monitoring with electronic BP machine with clinician intervention when threshold for intervention is met
**Control**	Clinic manual blood pressure monitoring
**Outcomes**	Maternal<br>1) Preeclampsia<br>2) Gestational hypertension<br>3) Maternal Mortality<br>4) Eclampsia, Stroke<br>5) Cortical blindness<br>6) Retinal detachment<br>7) Pulmonary oedema<br>8) Acute kidney Injury<br>9) Liver Capsule Haematoma or rupture<br>10) Placental abruption<br>11) post partum haemorrhage<br>12) Raised Liver enzymes<br>13) low platelet<br>14) admission into ICU<br>15) Intubation and mechanical ventilation<br><br>Fetus<br>1) Stillbirth<br>2) preterm delivery<br>3) small for gestational age<br>4) neonatal mortality<br>5) neonatal seizure<br>6) admission into NICU<br>7) Respiratory support

Literature search

Key words & search terms

Hypertensive disorders of pregnancy **OR** preeclampsia **OR** pre-eclampsia **OR** Gestational Hypertension **OR** Chronic Hypertension

AND

Home blood pressure monitoring **OR** Automated blood pressure monitor **OR** Remote blood pressure monitoring **OR** Electronic blood pressure machine

AND

Eclampsia **OR** Maternal mortality **OR** intra uterine growth restriction **OR** intra uterine growth retardation **OR** IUGR **OR** NICU admission **OR** ICU admission **OR** Stroke **OR** Cortical blindness **OR** Placental abruption **OR** Stillbirth **OR** Pulmonary oedema **OR** small for gestational age

Search range : Jan 1970 – Dec 2022

Exclusions : Non-human studies, Non-pregnant subjects

Platform : Cochrane, Medline, Embase, PubMed

- Records screened: 2,065
- Records excluded at title/abstract screening: 1,983
- Full-text reports assessed: 78
- Full-text reports excluded: 74
- Randomized trials included: 4

Studies included

Authors	Title	Study type
Ross-McGil 2000	Antenatal home blood pressure monitoring: a pilot randomised controlled trial	RCT
Tucker 2022	Effect of Self-monitoring of Blood Pressure on Diagnosis of Hypertension During Higher-Risk Pregnancy: The BUMP 1	RCT
Chappell 2022	Effect of Self-monitoring of Blood Pressure on Blood Pressure Control in Pregnant Individuals With Chronic or Gestational Hypertension: The BUMP 2 Randomized Clinical Trial	RCT
Pealing 2019	A randomised controlled trial of blood pressure self-monitoring in the management of hypertensive pregnancy. OPTIMUM-BP: A feasibility trial.	RCT

Studies excluded

Authors	Title	Reason for exclusion
De Greeff 2010	Accuracy assessment of the Dinamap ProCare 400 in pregnancy and preeclampsia	Device accuracy assessment. Not directly relevant to PICO
Brown 2018	Accuracy of automated blood pressure recorders in pregnancy	Device accuracy assessment. Not directly relevant to PICO
De Greeff 2009	Accuracy of inflationary versus deflationary oscillometry in pregnancy and preeclampsia: OMRON-MIT versus OMRON-M7	Device accuracy assessment. Not directly relevant to PICO
Gupta 2007	Accuracy of oscillometric blood pressure monitoring in pregnancy and pre-eclampsia	Device accuracy assessment. Not directly relevant to PICO
Hunter 2000	Accuracy of the Hewlett-Packard automated blood pressure monitor in pregnancy and pre-eclampsia	Device accuracy assessment. Not directly relevant to PICO
Reinders 2005	An accurate automated blood pressure device for use in pregnancy and pre-eclampsia: the Microlife 3BTO-A	Device accuracy assessment. Not directly relevant to PICO
Nathan 2015	An accurate semiautomated oscillometric blood pressure device for use in pregnancy (including pre-eclampsia) in a low-income and middle-income country population: the Microlife 3AS1-2	Device accuracy assessment. Not directly relevant to PICO
Aldrigde 2019	Agreement of aneroid and oscillometric blood pressure devices used in pregnancy	Device accuracy assessment. Not directly relevant to PICO
Roche 2018	Analysis of comparable home/clinic blood pressure readings in the LEANBH (Learning to Evaluate and manage ANtenatal Blood pressure at Home) population	Home blood pressure monitoring to identify white coat hypertension. Not directly relevant to this PICO

Brown 2004	Automated self-initiated blood pressure or 24-hour ambulatory blood pressure monitoring in pregnancy?	Device accuracy assessment. Not directly relevant to PICO
Penny 1998	Automated, ambulatory, or conventional blood pressure measurement in pregnancy: which is the better predictor of severe hypertension?	Device accuracy assessment. Not directly relevant to PICO
Rey 2009	Blood pressure assessments in different subtypes of hypertensive pregnant women: office versus home patient- or nurse-measured blood pressure	Comparison of accuracy between two assessment methods. Not directly relevant to PICO
Ishikuro 2013	Blood pressure measured in the clinic and at home during pregnancy among nulliparous and multiparous women: the BOSHI study	Assessment of accuracy of home BP monitoring. Not directly relevant to PICO due to difference in outcomes
Mooney 1990	Blood pressure measured telemetrically from home throughout pregnancy	Device accuracy assessment. Not directly relevant to PICO
Vigato 2019	Blood pressure measurement by oscillometric and auscultatory methods in normotensive pregnant women	Device accuracy assessment. Not directly relevant to PICO. Not in English
Green 1996	Blood pressure measurement during pregnancy: auscultatory versus oscillatory methods	Device accuracy assessment. Not directly relevant to PICO
Chohan 2016	Blood pressure profile in pregnancy: The impact of its duration on results and patients' well-being	Patient/consumer satisfactory assessment
Wagner 2013	Blood pressure self-measurement in the obstetric waiting room	Not directly relevant to PICO
Tucker 2017	Blood pressure self-monitoring in pregnancy: examining feasibility in a prospective cohort study	Not directly relevant to PICO
Swindells 1990	Blood pressure telemetry from home	Abstract only
Brown 2011	Can we use the Omron T9P automated blood pressure monitor in pregnancy?	Device accuracy assessment. Not directly relevant to PICO
De Greeff 2015	Clinical accuracy of a low cost portable blood pressure device in pregnancy and pre-eclampsia: the Nissei DS-400	Device accuracy assessment. Not directly relevant to PICO
Topouchian 2018	Clinical accuracy of the Omron M3 Comfortand the Omron Evolv for self-blood pressure measurements in pregnancy and pre-eclampsia - validation according to the Universal Standard Protocol	Device accuracy assessment. Not directly relevant to PICO
Hofstede 2019	A clinical evaluation of blood pressure measurement by iHealth BP5 in pregnancy	Device accuracy assessment. Not directly relevant to PICO
Davis 2015	Comparisons of auscultatory hybrid and automated sphygmomanometers with mercury sphygmomanometry in hypertensive and normotensive	Device accuracy assessment. Not directly relevant to PICO

	pregnant women: parallel validation studies	
Dalton 1987	Computerized home telemetry of maternal blood pressure in hypertensive pregnancy	Device accuracy assessment. Not directly relevant to PICO
Ochsenbein-Kolble 2004	Cross sectional study of automated blood pressure measurements throughout pregnancy	Device accuracy assessment. Not directly relevant to PICO
Nathan 2018	Early warning system hypertension thresholds to predict adverse outcomes in pre-eclampsia: A prospective cohort study	Not directly relevant to PICO
Taylor 2001	Evaluation of ambulatory and self-initiated blood pressure monitors by pregnant and postpartum women	Not directly relevant to PICO
Sheehan 2016	Evaluation of home monitoring of hypertension in pregnancy	Not directly relevant to PICO
Tucker 2018	How Do Home and Clinic Blood Pressure Readings Compare in Pregnancy?	Feasibility study
Veestgaard 2016	Home blood pressure in pregnancy-the upper reference limit	Not directly relevant to PICO
Lam 2017	Home blood pressure measurement in women with pregnancy-related hypertensive disorders	Prospective study
Naef 1998	Home blood pressure monitoring for pregnant patients with hypertension	Prospective study of 7 women (case study)
Kalafat 2020	Home blood pressure monitoring in the antenatal and postpartum period: A systematic review meta-analysis	SR
Perry 2018	Home blood-pressure monitoring in a hypertensive pregnant population	Case control study
Xydopoulus 2019	Home blood-pressure monitoring in a hypertensive pregnant population: cost-minimization study	Cost analysis study
Peek 1996	Hypertension in pregnancy: which method of blood pressure measurement is most predictive of outcome?	Comparison of methods of assessment
Lanssens 2018	The impact of a remote monitoring program on the prenatal follow-up of women with gestational hypertensive disorders	Retrospective study
Golara 2002	Inflationary oscillometry provides accurate measurement of blood pressure in pre-eclampsia	Comparison of devices
Kalafat 2018	Is home blood-pressure monitoring in hypertensive disorders of pregnancy consistent with clinic recordings?	Prospective cohort study
Brown 1993	Measurement of blood pressure during pregnancy: evaluation of the 'TriCUFF'	Comparison of BP cuff sizes

Dawson 1989	Miniature electronic blood pressure monitor compared with a blind-reading mercury sphygmomanometer in pregnancy	Comparison between devices
Mooney 1991	No difference between home and clinic blood pressure measurements in pregnancy: a computerized telemetric study	Case control study
Salazar 2019	Office blood pressure values and the necessity of out-of-office measurements in high-risk pregnancies	Prospective cohort study
Waugh 2003	Patient initiated home blood pressure recordings are accurate in hypertensive pregnant women	Feasibility study
Raichle 2018	Performance of a Blood Pressure Smartphone App in Pregnant Women: The iPARR Trial (iPhone App Compared With Standard RR Measurement)	Application validation study
Ohmaru 2012	The preceding features before onset of gestational hypertension and preeclampsia in home blood pressure monitoring: The existence of the inflection point and the rapid increased speed of blood pressure	Abstract only
Wagner 2014	Reliable blood pressure self-measurement in the obstetric waiting room	Not directly relevant to PICO
Ganapathy 2016	Remote monitoring of blood pressure to reduce the risk of preeclampsia related complications with an innovative use of mobile technology	Not directly relevant to PICO
van den Heuvel 2019	SAFE@HOME - Feasibility study of a telemonitoring platform combining blood pressure and preeclampsia symptoms in pregnancy care	Feasibility study
van den Heuvel 2020	SAFE@HOME: Digital health platform facilitating a new care path for women at increased risk of preeclampsia - A case-control study	Case control study
Pealing 2019	Self-monitoring blood pressure in hypertensive pregnancies: The OPTIMUM-BP pilot randomisedcontrolled trial	Duplicate
Lihme 2017	Self-monitoring of blood pressure during pregnancy: an observational study in the obstetrical waiting room	Prospective study. Not directly relevant to PICO
Tucker 2019	Self-monitoring of blood pressure during pregnancy: the BUMP trials	Protocol. Main study included
Bellomo 1995	Twenty four-hour ambulatory blood pressure monitoring in women with pre-eclampsia	Not directly relevant to PICO

Lo 2002	Use of automated home blood pressure monitoring in pregnancy: is it safe?	Validation of device
Chung 2009	Validation and compliance of a home monitoring device in pregnancy: microlife WatchBP home	Validation of device
van den Heuvel 2019	Validation of the iHealth Track and Omron HEM-9210T automated blood pressure devices for use in pregnancy	Device accuracy assessment. Not directly relevant to PICO
Clark 2018	Validation of the Microlife WatchBP Home blood pressure device in pregnancy for medium and large arm circumferences	Device accuracy assessment. Not directly relevant to PICO
Abbud 2018	Validation of the Omron HBP-1300 in pregnancy for medium-arm and large-arm circumferences according to the British Hypertension Society protocol	Device accuracy assessment. Not directly relevant to PICO
Nouwen 2012	Validation of the Omron M7 and Microlife 3BTO-A blood pressure measuring devices in preeclampsia	Device accuracy assessment. Not directly relevant to PICO
James 2017	Validation of the Omron MIT Elite blood pressure device in a pregnant population with large arm circumference	Device accuracy assessment. Not directly relevant to PICO
Kuper 2019	Validation of the SunTech Medical Advantage Model 2 Series Automated Blood Pressure Module in Pregnancy	Device accuracy assessment. Not directly relevant to PICO
Reinders 2003	Validation of the Welch Allyn 'Vital Signs' blood pressure measurement device in pregnancy and pre-eclampsia	Device accuracy assessment. Not directly relevant to PICO
Hay 2016	Validation of the Withings BP-800 in pregnancy and impact of maternal characteristics on the accuracy of blood pressure measurement	Device accuracy assessment. Not directly relevant to PICO
Kalafat 2019	Pregnancy outcomes following home blood pressure monitoring in gestational hypertension	Duplicate
Brown 2012	A prospective randomized study of automated versus mercury blood pressure recordings in hypertensive pregnancy (PRAM Study)	Device accuracy assessment. Not directly relevant to PICO
Mikami 2017	Provisional criteria for the diagnosis of hypertension in pregnancy using home blood pressure measurements	Not relevant to PICO
Butler Toba 2019	Randomized comparison of a reduced-visit prenatal care model enhanced with remote monitoring	Feasibility study
Rhodes 2018	A randomized trial of ambulatory blood pressure monitoring versus clinical blood pressure measurement in the management of hypertension in pregnancy. A feasibility study	Feasibility study

Tremonti 2017	Reliability of home blood pressure monitoring devices in pregnancy	Feasibility study
Al Hamarneh 2013	The validity of blood pressure kiosk validation studies: a systematic review	SR
Ashworth 2020	Setting and techniques for monitoring blood pressure during pregnancy<br>*** Data from this publicly published and available Cochrane Meta-analysis was referred to and adapted for this analysis*	SR
Bergel 2002	Ambulatory versus conventional methods for monitoring blood pressure during pregnancy<br>*** Data from this publicly published and available Cochrane Meta-analysis was referred to and adapted for this analysis*	SR
